# Hybrid Nanoplatforms Comprising Organic Nanocompartments Encapsulating Inorganic Nanoparticles for Enhanced Drug Delivery and Bioimaging Applications

**DOI:** 10.3390/molecules28155694

**Published:** 2023-07-27

**Authors:** Fatih Yanar, Dario Carugo, Xunli Zhang

**Affiliations:** 1 Department of Molecular Biology and Genetics, Bogazici University, 34342 Istanbul, Türkiye; 2 Nuffield Department of Orthopedics, Rheumatology and Musculoskeletal Sciences (NDORMS), University of Oxford, Oxford OX3 7LD, UK; dario.carugo@ndorms.ox.ac.uk; 3 School of Engineering, Faculty of Engineering and Physical Sciences, University of Southampton, Southampton SO17 1BJ, UK

**Keywords:** organic nanoparticles, inorganic nanoparticles, encapsulation, hybrid nanoparticles, drug delivery, bioimaging

## Abstract

Organic and inorganic nanoparticles (NPs) have attracted significant attention due to their unique physico-chemical properties, which have paved the way for their application in numerous fields including diagnostics and therapy. Recently, hybrid nanomaterials consisting of organic nanocompartments (e.g., liposomes, micelles, poly (lactic-co-glycolic acid) NPs, dendrimers, or chitosan NPs) encapsulating inorganic NPs (quantum dots, or NPs made of gold, silver, silica, or magnetic materials) have been researched for usage in vivo as drug-delivery or theranostic agents. These classes of hybrid multi-particulate systems can enable or facilitate the use of inorganic NPs in biomedical applications. Notably, integration of inorganic NPs within organic nanocompartments results in improved NP stability, enhanced bioavailability, and reduced systemic toxicity. Moreover, these hybrid nanomaterials allow synergistic interactions between organic and inorganic NPs, leading to further improvements in therapeutic efficacy. Furthermore, these platforms can also serve as multifunctional agents capable of advanced bioimaging and targeted delivery of therapeutic agents, with great potential for clinical applications. By considering these advancements in the field of nanomedicine, this review aims to provide an overview of recent developments in the use of hybrid nanoparticulate systems that consist of organic nanocompartments encapsulating inorganic NPs for applications in drug delivery, bioimaging, and theranostics.

## 1. Introduction

Nanoparticles (NPs) are submicroscopic particles with dimensions typically ranging between 1 and 100 nanometers (nm) in diameter. The unique physico-chemical properties of NPs, including their small size, large surface-to-volume ratio, and unique optical behaviour, make them a suitable candidate system for usage in the field of nanomedicine, especially in drug delivery and bioimaging applications. Examples of commonly employed NPs in nanomedicine include organic particulate systems, such as liposomes, micelles, dendrimers, poly (lactic-co-glycolic acid) (PLGA), and chitosan NPs, as well as inorganic NPs such as quantum dots (QDs) and NPs made of gold (AuNPs), silver (AgNPs), silica (SNPs), or magnetic materials (MNPs).

NP-based drug-delivery systems (DDSs) are designed for delivering a drug (or a combination of drugs) to a specific region within the body, in order to primarily cause damage to target cells whilst reducing side-effects due to systemic drug distribution in off-target regions. For example, it has been demonstrated that drug-encapsulating liposomes can enhance targeting and treatment efficiency compared with the free form of the drug [1]. Notably, DDSs have been successful in treating cancer, as well as a wide range of other diseases and conditions. For example, they have shown potential for improving treatment of infectious diseases [2], respiratory diseases [3], hypertension [4], diabetes [5], and for targeting the brain vasculature to enable drug transport across the blood–brain barrier [6].

Building upon the achievements of liposomal products, it has become increasingly apparent that NPs hold potential for overcoming widely recognized challenges such as those associated with the delivery of poorly water-soluble drugs, the transport of drugs across tight epithelial barriers, the intracellular delivery of large molecules, and the co-delivery of two or more drugs [7]. The range of applications has also extended beyond drug delivery to include detection of proteins, tissue engineering, tumour diagnosis, purification of molecules/cells, and biomedical imaging [8]. In recent years, the use of different types of NPs in nanomedicine has increased significantly, largely due to their ability to lower toxicity and improve bioavailability of therapeutic payloads, as well as for their applicability as contrast agents in biomedical imaging. 

Although organic and inorganic NPs individually are cornerstones of nanomedicine, these nanoparticulate systems can also be used in combination to achieve multifunctional features. These can be obtained via encapsulation or surface modification of the NPs, resulting in hybrid (organic–inorganic) nanoplatforms with enhanced diagnostic and/or therapeutic performance. Significant efforts have been dedicated to the development of hybrid nanoplatforms with a core–shell architecture where inorganic NPs are encapsulated within the aqueous core of organic nanocompartments, or where inorganic NPs are stabilised by an outer layer of organic compounds. These hybrid nanoplatforms have been evaluated for different applications [9,10,11], since the combination of organic/inorganic NPs is thought to elicit synergistic effects and to also improve the effectiveness and safety of inorganic NPs. 

**Figure 1:**
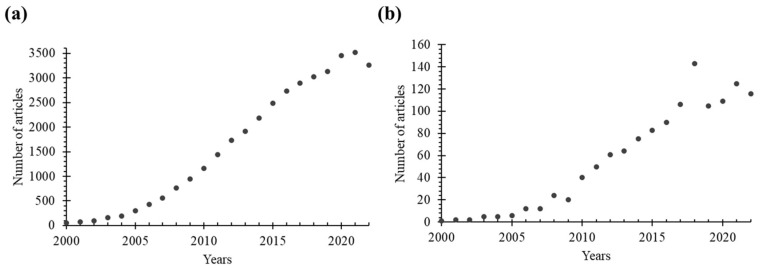
The number of research articles indexed in the Web of Science Core Collection (in Science Citation Index Expanded) was determined through a search using the following keywords: (**a**) “nanoparticle AND drug delivery” OR “nanoparticle AND imaging”, and (**b**) combinations of “hybrid”, “organic”, “inorganic”, and “nanoparticle” AND “drug delivery” OR “imaging” in the title or abstract.

Inorganic NPs have characteristics of large surface area and high reactivity, which can lead to agglomeration or degradation [12]. Moreover, they generally exhibit poor solubility in biological fluids, which can potentially trigger an immune response. In this context, the protection of inorganic NPs using an organic ‘shield’ can provide a physical barrier that prevents direct contact of inorganic NPs with biological structures, thereby reducing unwanted toxic effects. The presence of an organic shell or coating also improves the solubility, stability, and biocompatibility of inorganic NPs, while promoting or enabling targeted delivery and cellular uptake. Additionally, surface functionalization of inorganic NPs can allow application in targeted delivery and controlled release. Overall, the presence of an organic compartment (i.e., shell or coating) can allow and extend the use of inorganic NPs in drug delivery and biomedical imaging. As a result, hybrid organic/inorganic nanoplatforms have attracted significant attention in recent years and have been extensively studied in the literature. This is evident from Figure 1, which shows the number of published research articles specifically focusing on the use of such nanoparticulate systems in the fields of drug delivery and bioimaging.

This review presents a comprehensive overview of recent developments in this field of research, with a particular focus on nanoparticulate systems for enhanced therapeutic efficacy and real-time biomedical imaging. Specifically, this review covers the use of hybrid platforms (such as core–shell structures) that employ a variety of organic nanocompartments (such as liposomes, micelles, PLGA and chitosan NPs, and dendrimers) that encapsulate inorganic NPs (such as AuNPs, AgNPs, QDs, SNPs, and MNPs). These nanoscale systems represent promising nanodevices for drug delivery and bioimaging applications within the field of nanomedicine.

## 2. Organic Nanoparticles/Nanocompartments

**Figure 2:**
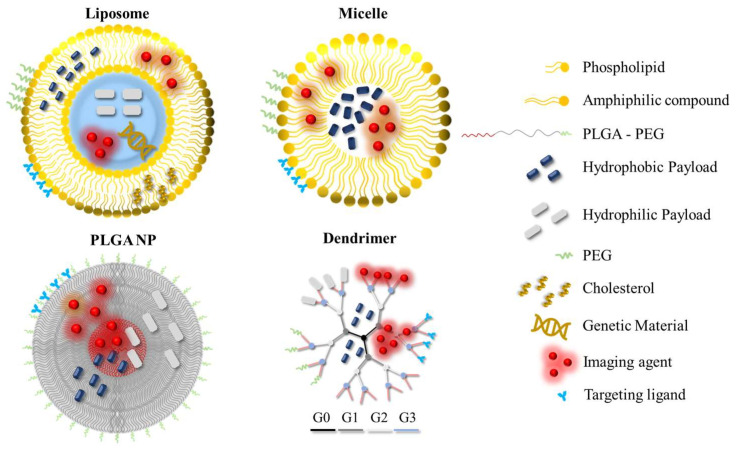
Organic nanoparticles that are commonly utilised in the field of nanomedicine. The nanoparticles can be modified by incorporating hydrophilic/hydrophobic drugs, PEG, targeting ligands or imaging agents for improved performance or added functionalities across different application areas. The different generations of the dendrimer are shown in different colours. PEG: polyethylene glycol; PLGA: poly (lactic-co-glycolic acid); G: generation number.

Organic NPs (or nanocompartments) are nanoparticulate systems composed of organic materials or compounds, and have been researched and utilised for a plethora of biomedical applications as drug nanocarriers or imaging probes. A schematic overview of organic NPs that are commonly employed in the field of nanomedicine is given in Figure 2.

Liposomes are composed of natural or synthetic lipids (typically phospholipids), and consist of lipid bilayers encapsulating aqueous compartments. They have characteristics of high biocompatibility and biodegradability, low toxicity, and the ability to encapsulate both hydrophobic and hydrophilic molecules [13,14]. Together, those features make liposomes highly attractive as nanocarriers for drug-delivery applications. Liposomes can be synthesized in different sizes, ranging between 50 and 1000 nm, using different techniques such as thin-film hydration, reverse-phase evaporation, lipid extrusion, or microfluidics [14,15].

The size of NPs has great importance in drug-delivery applications as it determines their circulation half-life and drug encapsulation efficiency, and also influences their ability to extravasate across the tumour vasculature (i.e., through the so-called enhanced permeability and retention (EPR) effect) as well as their recognition by macrophages [16]. It has been reported that liposomes with size greater than 100 nm can be easily identified by macrophages, leading to accumulation in organs containing the mononuclear phagocyte system, such as the liver and spleen. NPs smaller than 10 nm can be rapidly cleared by the renal excretion system, considering that the size of the average renal filtration pore is of around 10 nm. NPs can also be subject to rapid clearance due to opsonization by serum proteins, whereby liposomes become recognizable by the immune system and can be digested by macrophages of the reticuloendothelial system (RES) [17], especially in the liver and spleen, resulting in lowered therapeutic efficacy overall. To overcome this challenge, a hydrophilic polymer, polyethylene glycol (PEG), has been widely employed to coat the surface of NPs to improve their stability and circulation time [18]. The steric barrier provided by PEG mitigates the extent of opsonization and recognition by macrophages, thus resulting in increased accumulation at the intended site of treatment and reduced side-effects [19].

Liposomes are generally utilised as drug-delivery vehicles, mainly due to the versatility offered by their architecture that makes them suitable for incorporation of both lipophilic and hydrophilic pharmaceutical actives within their lipid bilayer and aqueous core, respectively. Liposomal products are able to deliver drugs to a specific site within the body while minimizing the negative side-effects of systemic exposure, since the pharmaceutical agent is confined by the liposome membrane and therefore healthy tissues are not directly exposed to it. Moreover, the liposome surface can be functionalized with targeting ligands, antibodies, peptides, and/or imaging moieties for application in diagnostics, therapy, or the concurrent combination of these (often referred to as ‘theranostics’).

Micelles consist of closed monolayered structures typically formed by the aggregation of amphiphilic compounds into stable ordered units, by self-assembly above a critical micelle concentration (CMC) [20]. The structure of micelles is characterized by a fatty acid core with a polar surface, or by a polar core with fatty acids on the surface which is referred to as an ‘inverted micelle’ configuration [21]. Preparation methods of micelles include solvent evaporation [22], dialysis [23], and direct dissolution [24]. The size of micelles typically ranges between 10 and 100 nm, and various shapes can be synthesized depending on the specific application of interest [20]. Polymeric micelles are a type of micelle formed by amphiphilic block copolymers (composed of hydrophobic and hydrophilic blocks), and are typically characterized by a larger volume, lower CMC, and greater stability. The structure of micelles makes them suitable for encapsulation of hydrophobic bioactive compounds within their core, while the outer hydrophilic layer provides stability and protection from recognition by RES. Thus, micelles are a suitable candidate system for improving solubility, stability, and bioavailability of hydrophobic payloads. The surface of micelles can also be functionalized using targeting moieties such as antibodies or peptides, providing the ability to bind to specific receptors on target cells. In this context, micelles can be employed in a number of biomedical applications including drug delivery, extraction of proteins, and bioimaging [25,26]. 

Poly (lactic-co-glycolic acid) (PLGA) is a widely used polymer in the field of nanomedicine, especially due to its remarkable biodegradability and biocompatibility. It is composed of two monomeric units of lactic acid and glycolic acid, which can be readily metabolized with minimal toxicity [27]. The structure of PLGA can be designed by adjusting the relative amount and/or the molecular weight of these monomers, facilitating the tailored synthesis of NPs towards specific applications. The synthesis of PLGA NPs can be performed by nanoprecipitation or emulsification–evaporation techniques [28]. Depending on the application and the desired encapsulation performance, single (oil-in-water) or double (water-in-oil-in-water) emulsion templating techniques can be utilised [27]. The size of PLGA NPs can vary between 10 and 1000 nm [29], and it can be regulated by controlling experimental conditions such as stirring rate and temperature [30]. PLGA NPs are suitable for encapsulating hydrophilic and/or hydrophobic molecules, while the surface can be modified with various moieties for reduced toxicity, enhanced stability, targeted delivery, or theranostic functionalities. PLGA NPs are especially considered for sustained drug release, since their controllable properties enable the design of desirable drug-release profiles, overall making them ideal nanocarriers for treatments demanding long-term therapy with reduced dosing frequency.

Dendrimers are characterized by a well-defined tree-like structure with a central core and multiple layers of branch units on the outer shell. Branches originate from the central core and their number and branching architecture can be designed based on the target application. The architecture of dendrimers allows precise control over their size and shape, as well as the functionalization of their surface. The most commonly employed techniques for the synthesis of dendrimers are divergent and convergent methods [31]. The size of dendrimers typically ranges between 1 and 15 nm; however, high-generation dendrimers can have larger sizes [32]. Dendrimers have a high loading capacity for pharmaceutical agents and can provide sustained drug-release profiles. They can be functionalized with imaging probes or contrast agents, making them suitable for diagnostic imaging. Moreover, they can be employed in a number of other applications such as gene delivery, tissue engineering, and catalysis. Polyamidoamine (PAMAM) dendrimers represent one of the most utilised families of dendrimers due to their unique physico-chemical properties, such as high water solubility, biocompatibility, and precise structural control, overall making them suitable for therapeutic and diagnostic applications [33].

Chitosan is a deacetylated form of chitin, which is derived from crustacean shells or the cell walls of fungi. It is a widely used, FDA-approved, biodegradable, biocompatible polymer and is often utilised as a nanocarrier material in drug-delivery applications. Various methods have been proposed for the synthesis of chitosan NPs, such as microemulsification, emulsification–solvent diffusion, emulsion-based solvent evaporation, and ionotropic gelation [34]. Beyond application in drug delivery, these NPs have demonstrated potential for application in tissue engineering and as antimicrobial or antioxidant agents. As for other nanoparticulate systems, the surface of chitosan NPs can also be modified for targeting or imaging purposes. Moreover, chitosan NPs are known for their mucoadhesive and sustained drug-release properties, making them an ideal candidate system for mucosal drug-delivery applications [34]. 

## 3. Inorganic Nanoparticles

**Figure 3:**
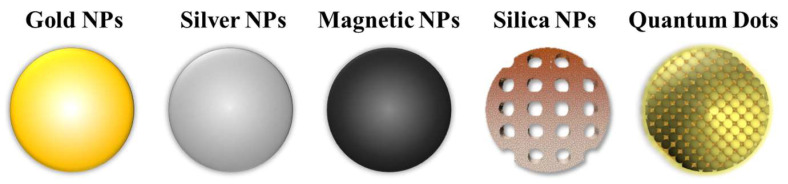
Examples of inorganic nanoparticles that are commonly employed in nanomedicine, which include: gold, silver, magnetic, and silica NPs, and quantum dots.

Inorganic NPs are nanoscale particulate systems composed of inorganic materials such as metals or semiconductors. They have unique optical properties due to their small size and high surface-to-volume ratio, which make them valuable tools for application in drug delivery, biomedical imaging, and theranostics [35]. Examples of inorganic NPs utilised in the field of nanomedicine are shown in Figure 3.

Gold NPs (AuNPs) and silver NPs (AgNPs) have attracted significant attention in industry, and in technological development more broadly, especially due to their remarkable optical properties. Notably, these NPs can display surface plasmon resonance (SPR) behaviour, which occurs when light excites electrons at the interface between the conductor and insulator components of the material [36]. The optical properties of these NPs are mainly shape- and size-dependent, and accordingly, the SPR also depends on the composition, shape, and size of the NPs [37,38]. When light is absorbed on the surface of metal NPs, electrons undergo a collective oscillation, which generates local heat that can be utilised in biomedical applications. In principle, when plasmonic NPs, such as gold or silver, are encapsulated inside an outer layer, they can cause this layer to phase transition when they are subjected to light irradiation as they can convert optical energy into local heat energy [39]. It is important to note that this phenomenon also enables photothermal therapy (PTT), which refers to the utilization of light energy to kill cancer cells [40]. Various physico-chemical methods for the synthesis of AuNPs and AgNPs have been reported in the literature, including laser ablation, chemical reduction, and green synthesis [41]. The size and shape of the NPs are highly dependent on the production technique employed. AuNPs and AgNPs are also attractive for drug-delivery applications, as they can be designed to function as controlled drug-release systems. This feature of NPs relies on the photothermal effect (PTE), which induces the release of encapsulated payloads through an outer layer surrounding the NP, due to a phase change in the layer caused by localized temperature increases, as described above. In addition, among metallic NPs, AgNPs are widely used in healthcare products and in the food industry due to their remarkable antibacterial properties.

Quantum dots (QDs) are semiconductor nanomaterials composed of groups II-VI, III-V, or IV-VI elements [42]. Synthesis methods of QDs are classified into two main categories: top-down and bottom-up approaches. In top-down approaches, a semiconductor is thinned down to form QDs, while in bottom-up approaches, a self-assembly process is performed to create QDs [43]. These NPs have a small size of around 10 nm and possess unique optical and electronic properties arising from quantum confinement effects. Their tuneable emission wavelength makes them ideal agents for various applications in bioimaging, detection, and tracking. Notably, QDs have an absorption wavelength higher than 650 nm in the near-infrared (NIR) region, which is advantageous in biomedical imaging applications as it presents minimal tissue absorption [44]. In drug-delivery applications, the surface of QDs can be functionalized with phospholipids or amphiphilic polymers to create a hydrophilic protective shell. This can improve the stability and biocompatibility of QDs in biological fluids, enabling their utilization in biomedical applications, e.g., for improving treatment efficacy. It can also provide steric stabilization, reduce non-specific interactions, and enhance circulation time within the body. Overall, QDs are particularly attractive for improving imaging contrast, detection sensitivity of biological targets, targeted drug delivery, and theranostics.

Silica NPs (SNPs) are composed primarily of silicon dioxide (SiO_2_) and their size typically ranges between a few nm to a few hundred nm. Commonly employed techniques for the synthesis of SNPs include the microemulsion method and the Stöber process. The structure of SNPs can be adapted to achieve various architectural shapes including spheres, rods, or tubes [45]. They have characteristics of high stability and biocompatibility, making them suitable nanomaterials for a range of biomedical applications. The surface of SNPs can be functionalized with specific molecules allowing targeted delivery, controlled release, or contrast enhancement in bioimaging. The porous structure of SNPs also enables high drug-loading capacity and efficient delivery of pharmaceutical agents. SNPs can be engineered to incorporate fluorescent dyes, QDs, or other imaging agents. This property allows them to be utilised as imaging probes for fluorescence imaging, magnetic resonance imaging (MRI), or computed tomography (CT). For this reason, SNPs can serve as effective platforms in biosensing applications, including for the detection of biomarkers or for ‘tracking’ biological processes dynamically [46].

Magnetic NPs (MNPs) refer to particles in the nanoscale that possess magnetic properties. These particles are typically composed of a magnetic core material (e.g., iron oxide), coated with a functional shell [47]. The core of MNPs is responsible for their magnetic behaviour. Iron oxide magnetic NPs (IONPs) are commonly used as MNPs due to their high magnetization and biocompatibility. Their small size (up to a few hundred nm) provides benefits such as large surface-area-to-volume ratio, improved colloidal stability, and enhanced magnetic response. Among various methods for the synthesis of MNPs, the sol–gel method, coprecipitation, microemulsification, and thermal decomposition are commonly employed [48]. MNPs can be surface-modified with biocompatible coatings to enhance stability and biocompatibility; this makes them suitable for various biological and biomedical applications. They are commonly employed in MRI as contrast agents to enhance imaging resolution and sensitivity. Interestingly, MNPs can also be employed to enable magnetic hyperthermia, which is a therapeutic approach where NPs generate heat in response to an alternating magnetic field, leading to localized tumour ablation [49]. They can also be employed in targeted DDSs, biosensing, and magnetic cell separation. Thanks to the magnetic properties of these NPs, they can be controlled using external magnetic fields to localize in specific regions of the body, i.e., for targeted drug delivery or imaging. 

## 4. Properties of Nanoparticles with Encapsulation

**Figure 4:**
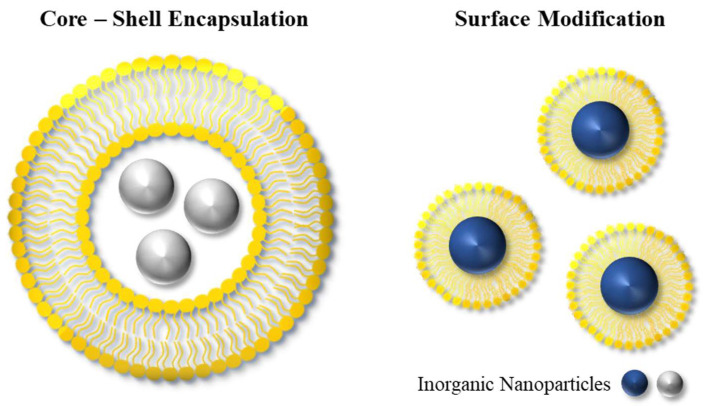
The design of organic–inorganic hybrid platforms can have various architectures, including those based on core–shell structures and surface modifications.

As discussed earlier, efficient delivery and ‘shielding’ of inorganic NPs through encapsulation can significantly enhance their performance in a range of biomedical applications. The advantages that the encapsulation process introduces include improved stability and biocompatibility, as well as controlled drug release and targeted drug-delivery capabilities. This can typically be achieved by encapsulating the NPs within a shell or modifying the surface of the NPs, as shown in Figure 4. Additionally, other types of hybrid NPs have been investigated in the literature, including hybrid nanogels or Janus particles [50]. Examples of methods for the encapsulation of inorganic NPs include the polymerization of the organic NPs around inorganic NPs, and the encapsulation via adsorption of oppositely charged particles onto inorganic NPs [35].

Typically, a polymeric nanocompartment or layer is created around the NPs, which can be achieved by heterogeneous polymerization techniques or through nanoprecipitation [51,52,53]. On the other hand, the surface modification method involves attaching organic molecules to the surface of the NPs, enhancing their functionality without creating an outer shell structure. Depending on the desired application, the surface chemistry of NPs can be tailored by selecting appropriate molecules to meet specific requirements. 

The efficiency of a given encapsulation method depends on various physico-chemical properties of the NPs, including their size, surface chemistry, surface charge, and solubility, as well as the desired characteristics of the hybrid nanoplatform, such as stability, biocompatibility, and controlled release and/or targeted delivery capabilities.

## 5. Biomedical Applications of Hybrid Nanoparticles

**Figure 5:**
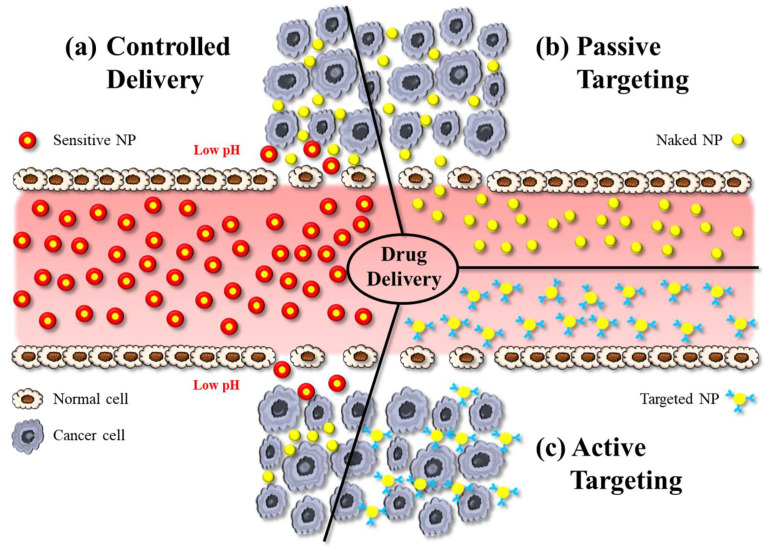
The illustration shows various approaches in nanoparticle-based drug delivery: (**a**) controlled delivery, (**b**) active targeting, and (**c**) passive targeting. NP: nanoparticle.

Hybrid NPs have been utilised as powerful tools in biomedical applications, especially for targeted drug delivery, bioimaging, and theranostics. Concerning applications in drug delivery, hybrid NPs—like other nanomedicines—can deliver drugs by active or passive targeting. Active targeting typically involves some modification on the particle surface, e.g., using charged lipids, antibodies, or attachment of ligands that enable the NP to bind to the receptors of the target cells and to cross biological membranes more effectively (Figure 5c). Active targeting therefore reduces undesired side-effects of drugs, while providing high therapeutic efficacy by allowing high dosing at the diseased site [54].

Passive targeting is the accumulation of NPs at pathological sites due to the EPR effect, whereby the increased vascular permeability enables enhanced extravasation of NPs and drugs (Figure 5b). Notably, the size of intercellular gaps in the vascular endothelium of these pathological regions increases by about 1 μm after exposure to inflammatory mediators. This helps NPs, for example, at tumour sites much more effectively than in physiological tissues [55]. The delivery of the payload to cells occurs via the interaction between the NP and the cell membrane, which can occur through different mechanisms. NPs can indirectly enter the cytoplasm by endocytosis, resulting in the delivery of the payload. An alternative process is fusion, whereby NP layers merge with the cell membrane, resulting in direct delivery of the payload. Another mechanism of interaction between NPs and cell membranes is lipid exchange, which involves the exchange of bilayer materials between the NP layers and the cell membrane. It is important to note that these interactions can trigger the immune system. Therefore, it is crucial to develop the surface chemistry of NPs in such a way to make them unrecognizable by the RES [17].

Controlled drug delivery, on the other hand, refers to the regulation of the release of a payload from a nanocarrier system (Figure 5c). These systems are designed to initiate drug release in response to an exogenous or endogenous trigger such as pH, temperature, or light. Sustained drug-delivery systems on the other hand provide prolonged release of a drug over a certain period of time. In DDSs designed for sustained release, the drug is typically encapsulated within the NP or a matrix system, and drug release occurs via diffusion [56].

Another important biomedical application of NPs is in biomedical imaging, i.e., as contrast agents. This approach has the potential to provide more accurate information about a given disease condition compared with traditional clinical imaging. Owing to the aforementioned targeting capabilities and the small size of NPs, nanoparticulate contrast agents can be directed to the targeted area, thus enabling accurate detection and imaging of a biological target tissue. This approach can be applied in fluorescence imaging, magnetic resonance imaging (MRI), computerized tomography (CT), ultrasound (US), and multimodal imaging [57]. 

Fluorescence imaging often involves the use of fluorophores or fluorescent dye molecules attached to NPs. These compounds can absorb light at specific wavelengths and emit light at longer wavelengths, when excited by an appropriate light source. Fluorophores can be targeted to specific cell types or cell structures for various purposes including protein analysis, gene detection, diagnostics, and real-time monitoring [58]. Fluorescence imaging can provide vital information, especially when NIR light is utilised, due to improved tissue penetration of light and reduced autofluorescence, allowing for enhanced imaging sensitivity. Fluorescence imaging can be implemented by conjugating fluorescent dyes with NPs or by encapsulating fluorescent agents within NPs.

MRI is one of the most commonly applied methods for disease diagnosis and monitoring in the clinic for various medical conditions. The fundamental principle of MRI is based on the movement of protons within a strong magnetic field. This provides high-resolution images of internal body structures in multiple planes. Commonly employed contrast agents in MRI include gadolinium-based NPs and superparamagnetic materials, due to their remarkable magnetic properties [59]. These contrast agents help enhance the visibility of targeted areas, improving the accuracy of the diagnostic process.

US imaging is also one of the most commonly employed methods for medical imaging, due to its simplicity of usage, safety, and real-time imaging capabilities. In this approach, sound waves (with a frequency >20 kHz) are generated by an extracorporeal transducer positioned in contact with the body. As these waves penetrate tissues, they encounter biological structures with different acoustical properties. These differences in properties between structures (i.e., mainly in density and compressibility) result in reflections of the ultrasound wave, which are captured by a probe and are then converted into images [60]. Different types of contrast agents are used in US imaging in order to enhance the acoustical properties mismatch between the vasculature and surrounding tissues; these include gas-filled (e.g., microbubbles with a core of a heavy gas, like perfluorocarbon), solid-based (e.g., silica NPs), and liquid-based (e.g., perfluorooctyl bromide) particles. Some of these NP systems present a core–shell design configuration. It is important to note that the aforementioned imaging approaches primarily focus on providing single imaging modalities; however, advancements have allowed the development of multifunctional NPs serving as imaging agents with multimodal imaging capabilities. These NPs can incorporate single (e.g., silicon naphthalocyanine) [61] or multiple imaging agents, enabling the simultaneous use of distinct imaging modalities [62]. This approach allows complementary information to be captured from different imaging techniques, thereby also improving accuracy and reliability of diagnosis.

**Figure 6:**
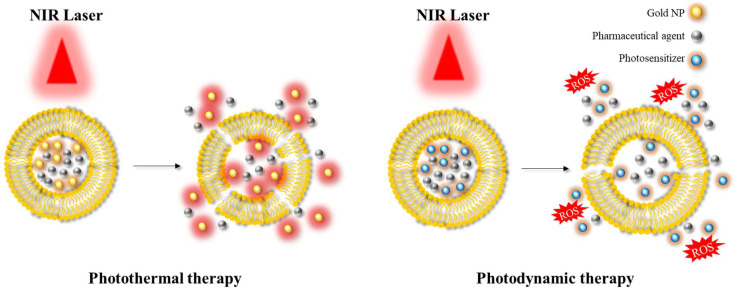
Illustration depicting the application of photothermal therapy and photodynamic therapy. NIR: near-infrared; ROS: reactive oxygen species.

Another significant area of application is photoablation therapy, which can be classified into two main modalities: photodynamic therapy (PDT) and photothermal therapy (PTT) [40,63] (Figure 6). PDT involves the use of (initially) non-toxic compounds called photosensitizers, which are exposed to light at a specific wavelength (e.g., in the NIR) resulting in the formation of toxic compounds. These toxic products can react with hydroxyl ions or water, and in turn form reactive oxygen species (ROS) that can cause cell death. This approach is mainly employed in the context of anticancer therapy, as the production of ROS can induce suppression of tumour growth. In PTT, the targeted area is irradiated using a light source at a specific wavelength similar to PDT. This light energy is converted into heat energy by specific materials, such as AuNPs or AgNPs, resulting in hyperthermia and cell death. PDT and PTT are examples of common therapeutic approaches utilising hybrid platforms.

### 5.1. Liposomes-Based Hybrid Platforms

Liposomes are one of the most commonly used nanocarriers for integration into hybrid platforms in nanomedicine. These platforms combine the unique advantages of liposomes and inorganic NPs, offering multi-functional features. Examples of studies that focused on hybrid platforms consisting of liposomes as the organic nanocompartment and inorganic NPs as the core are given in Table 1.

Xing et al. demonstrated the implementation of PTT by employing a liposomal system encapsulating doxorubicin (Dox), i.e., a chemotherapeutic compound and AuNPs as inorganic NPs [40]. When the hybrid platform was irradiated by NIR light, the liposome layers became permeable to both Dox and AuNPs, allowing the release of both payloads. It was reported that this platform resulted in effective tumour suppression with a cell growth inhibition rate of up to 78.28%. In a similar study, Koga et al. utilised AuNPs to achieve controlled drug release via PTE. Their results showed that the hybrid system was capable of releasing >80% of the drug in less than 1 min upon NIR irradiation [64]. Another approach, investigated by Lv et al., employed thermosensitive liposomes encapsulating Au nanorods, MNPs, and Dox for targeted delivery assisted by PTE [74]. This platform showed superparamagnetic properties and enabled controlled release, with approximately 95% of the drug released after 3 h of irradiation using a 980 nm laser beam. This approach was found to be highly effective in treating bladder tumour cells. Among many studies investigating liposome–AuNP hybrid platforms as DDSs, Li et al. demonstrated that incorporating hollow AuNPs in liposomes resulted in greater efficacy both in terms of the hyperthermia achieved within the targeted tissue and the drug (Dox) release profile, compared with liposomes loaded with solid AuNPs [72]. It is worth mentioning that, in addition to employing chemotherapeutic agents (such as Dox) as encapsulated payloads in liposomes for targeted and controlled drug-delivery applications, various other types of molecules have also been encapsulated in nanocompartments, including miRNA inhibitors [66] and fish oil protein [65]. Grafals-Ruiz et al. proposed a liposomal system for the treatment of glioblastoma (GBM), one of the most common types of brain tumours [66]. The system comprised liposomes encapsulating AuNPs functionalized with oligonucleotide miRNA inhibitors. In addition, the liposomes were conjugated to either apolipoprotein E (ApoE) or rabies virus glycoprotein. The hybrid nanoplatform was investigated in GBM syngeneic mice by intravenous administration. Results showed that the expression of miRNA-92b was effectively inhibited. Furthermore, compared with controls, liposomes conjugated with ApoE accumulated to a greater extent at the tumour tissue, suggesting improved targeted delivery.

Liposomal platforms have also been employed for enhanced bioimaging applications. A hybrid nanoplatform consisting of liposomes encapsulating AuNPs, perfluorocarbon, and Dox has been utilised for image-guided PTT [68], with positive outcomes in terms of both bioimaging and drug delivery. Another study conducted by Prasad et al. synthesized liposomes encapsulating AuNPs along with emissive graphene QDs for application in in vivo bioimaging and NIR-mediated cancer therapy [73]. The liposomes also encapsulated Dox as a chemotherapeutic drug, and their surface was functionalized with folic acid (FA) as a targeting ligand. The developed theranostic system demonstrated capability for in vivo bioimaging of tumour tissue using NIR light (wavelength of 750 nm). Moreover, it offered PDT and chemotherapeutic performance. Notably, NIR light exposure resulted in the generation of ROS, resulting in tumour reduction. 

In a similar theranostic approach by Li et al., perfluorocarbon was encapsulated in liposomes by film dispersion, with the aim of developing US contrast agents that are more effective at penetrating into a target tissue than conventional, micrometer-sized contrast agents [68]. This hybrid system also encapsulated Dox along with hollow AuNPs, to achieve PTT upon exposure to NIR light (wavelength of 808 nm). In vivo fluorescence imaging demonstrated the accumulation of NPs at the targeted area in a 4T1 tumour model. NIR illumination also resulted in localized hyperthermia leading to significant Dox release. The nanoplatform was also found effective at performing US image-guided PTT and chemotherapy. Charest et al. developed a liposomal formulation of AuNPs with carboplatin and evaluated its radiosensitizing potential [67]. The study found that simultaneous administration of low doses of carboplatin and AuNPs through encapsulation in liposomal nanocarriers resulted in effective radiosensitization.

The investigation of liposomal encapsulation of QDs, MNPs, and SNPs was also conducted for enhanced therapeutic efficacy. Chen et al. examined liposomes encapsulating both CdSe QDs modified with oleic acid and superparamagnetic iron oxide NPs (SPIONs), for the treatment of hepatocellular carcinoma [76]. This strategy led to the synthesis of magnetic fluorescent liposomes with multifunctional properties. The platform could label and image cancer cells with high biocompatibility, suggesting that it has the potential for improved targeted drug delivery. A study by Sun et al. demonstrated the capabilities of liposomes encapsulating MSNs, for triple-modal image-guided cancer therapy as a theranostic drug-delivery platform [80]. In this research, gadolinium-doped MSNs were encapsulated in liposomes along with Dox. Liposomes were coated with FA to prevent the leakage of Dox and for achieving targeted delivery. The nanoplatform was also conjugated with indocyanine green (ICG) to enable triple-modal imaging through NIR irradiation. Results demonstrated that ICG enabled PTT and PDT, while allowing NIR fluorescence imaging and photoacoustic imaging (PAI). The addition of gadolinium also enabled MRI capabilities. In vitro and in vivo studies showed improved antitumour effects with good imaging contrast, suggesting that this theranostic platform is a candidate for image-guided phototherapy. Overall, the system was successful at providing triple-modal imaging in a single platform, capable of NIR fluorescence imaging, PAI, and MRI.

### 5.2. Micelle-Based Hybrid Platforms

Micelles are widely used as organic NPs in biomedical applications, acting as nanocompartments through the creation of shells or coatings, as well as for encapsulating inorganic NPs. Some example biomedical applications of such micelle-based hybrid platforms are given in Table 2. 

Volsi et al. studied the design of a theranostic micellar nanoplatform for targeted cancer therapy [86]. The polymeric micellar structure consisted of α,β-poly(N-hydroxyethyl)-DL-aspartamide functionalized with lipoic acid (LA), PEG as a hydrophilic moiety, and FA as a targeting moiety which was able to self-assemble in aqueous solution. The platform also encapsulated Dox and Au core–shell QD NPs. Experiments showed that the nanocompartment was stable and efficient at targeting/delivering Dox to MCF7 cells, as well as capable of exploiting heat generation by PTE of QD-Au NPs. It was suggested that this theranostic hybrid system has potential for cancer treatment considering its enhanced drug-delivery behaviour and imaging capabilities, which can assist in diagnostics and therapy monitoring purposes. 

Micellar nanocompartments were also examined by Li et al. for pH-sensitive delivery and MRI imaging [90]. Researchers synthesized poly(ethylene glycol)-b-poly(β-benzyl L-aspartate) and aminolyzed it with N,N-diisopropylamino ethylamine and N,N-dibutalamino ethylamine at different molar ratios, for the development of an amphiphilic block copolymer capable of encapsulating SPIONs and Dox. The system was designed to encapsulate its payloads, i.e., drugs or contrast agents, under neutral pH conditions, providing stability and preventing premature release of payloads during circulation or storage. The nanoplatform was also designed to provide triggered release in weak acidic environments, typically found in certain pathological conditions. Experiments demonstrated effective uptake by HepG2 cells and successful release of Dox at low pH conditions, demonstrating the nanoplatform’s potential for therapeutic purposes. In addition, fluorescence and MRI studies revealed that the weak positive charge of the hybrid system contributed to longer blood circulation in vivo. The system thus exhibited successful pH-sensitive tumour targeting with efficient and non-invasive MRI visibility, allowing improved non-invasive image-guided therapy. Furthermore, it had a minimal side-effects profile, while displaying impressive anticancer outcomes. Further studies focusing on the applicability of micellar nanocompartments for enhanced tracking and biomolecular detection have been also carried out by encapsulating QDs and SPIONs [87].

### 5.3. PLGA-Based Hybrid Platforms

PLGA-based hybrid systems have recently emerged as promising platforms, especially for drug delivery and bioimaging applications. Table 3 shows selected publications covering PLGA-based hybrid platforms utilised in biomedical applications. 

Luo et al. utilised PLGA NPs for the encapsulation of anti-PD-1 peptide and hollow Au nanoshells for improved immunotherapy achieved by PD-1 blocking combined with PTT [91]. This hybrid system demonstrated long-term activation of the immune system over 40 days, which could also be accelerated using NIR laser illumination. It was also revealed that multiple irradiations using NIR laser illumination enhanced the antitumour effect, resulting in the inhibition of primary tumours as well as distant tumours. The therapy was also capable of enhancing immune cell activation. 

In another study conducted by Galliani et al., PLGA-based hybrid platforms were designed to implement PDT for enhanced cancer therapy [63]. Chlorophyllin–copper complex and CdSe/ZnS core–shell QDs were encapsulated successfully in the PLGA nanocompartments. Irradiation at 365 nm by UV resulted in the generation of ROS due to fluorescence resonance energy transfer between QDs and chlorophyllin. It was indicated by the authors that this platform has potential for PDT as it could generate ROS upon irradiation; however, further analysis is required to assess the underlying mechanisms and optimize the formulation.

As reported by Jin et al., a PLGA-based nanoplatform was designed for multimodal imaging and US-triggered drug delivery. SPIONs and Dox were successfully encapsulated in PLGA-based nanocompartments conjugated with PEG and FA [94]. In vitro experiments demonstrated the potential for US and MRI contrast imaging, as well as increased targeting ability due to FA conjugation. Focused US was utilised as a remote-control technique to trigger Dox release and induce cell membrane permeabilization. These findings highlighted the promising application potential of this system as a tool for US- and MRI-guided drug delivery in anticancer therapy.

Kumar et al. aimed to develop implantable nanoplatforms composed of PLGA-based nanocompartments encapsulating docetaxel and Cy7.5 (fluorophore) conjugated with SNPs [95]. In vivo studies demonstrated efficient sustained drug release near tissues. The docetaxel-loaded spacers exhibited suppression of tumour growth compared with the control over 16 days, demonstrating improved therapeutic efficacy. It is important to note that this system was also suitable for contrast imaging due to its fluorescent moiety (Cy7.5), with potential for use in disease monitoring. 

### 5.4. Dendrimer-Based Hybrid Platforms

Table 4 represents a selection of recently published articles focusing on the utilization of dendrimer-based hybrid platforms in biomedical applications. 

In a study by Ghosh et al., a dendrimer-based hybrid platform was utilised for targeted gene delivery for the treatment of triple-negative breast cancer (TNBC) [97]. The authors successfully synthesized carbon QDs conjugated with PAMAM dendrimers of different generations. RGDS peptides were further conjugated to the nanoplatform to be able to target the αvβ3 integrin, which is known to be overexpressed in TNBC. Among different conjugates, QD–PAMAM conjugate 3 showed superior capabilities for gene complexation and protection against enzymatic digestion. Furthermore, it exhibited efficient detection of Cu (II) ions, with a fluorescence quenching efficiency of 93%. It is important to note that TNBC often has higher levels of Cu (II) ions, which offers potential for the detection of the metastatic phase of TNBC.

In another approach, a pH-sensitive and self-fluorescent nanoplatform was developed using mesoporous SNPs and PAMAM dendrimers [99]. It was found that the inclusion of PAMAM dendrimers provided improved encapsulation efficiency and additional eligible reaction sites for modifications. In addition, the structure of PAMAM dendrimers affected the drug-release performance and prevented burst release. The fluorescence behaviour of this hybrid system offers the capability for potential biological tracking and bio-detection. Importantly, PAMAM dendrimers served as both pH-sensitive capping agents and self-fluorescent agents, possessing multiple functions in a single platform. This research demonstrated the versatility of dendrimer-based systems for developing multifunctional and biocompatible drug-delivery platforms for biomedical imaging, diagnosis, and simultaneous therapy. 

Dendrimer-based nanocompartments have also been utilised for the application in multimodal image-guided cancer therapy [100]. A theranostic nanoplatform was developed, comprising generation 5 poly(amidoamine) dendrimer-stabilized AuNPs embedded with ultrasmall IONPs, capable of MR/CT/PAI-guided PTT and radiotherapy (RT). This multifunctional nanoplatform induced significant cell death under laser irradiation. In vivo experiments demonstrated accumulation within the tumour tissue along with MRI, CT, and PAI imaging enhancement capabilities. These findings suggest that this nanoplatform has the potential for image-guided cancer therapy, leading to improved diagnosis and treatment while minimizing side-effects.

### 5.5. Chitosan-Based Hybrid Platforms

Chitosan has also been used as a constitutive material for hybrid platforms in biomedical applications. Examples of recently published studies focusing on chitosan-based hybrid platforms are given in Table 5.

Liu et al. developed a theranostic platform by incorporating cysteine functionalized AuNPs into chitosan/tripolyphosphate NPs (modified with polyacrylic acid), aiming for improved cellular uptake, high loading capacity, controlled release, and efficient bioimaging [101]. This hybrid platform was also loaded with Dox as the chemotherapeutic agent. In vitro experiments showed sustained drug release for up to 48 h under acidic conditions; however, drug release was accelerated at higher pH values. The hybrid platform also showed greater cellular uptake compared to free Dox. In vivo studies revealed that the drug accumulation could be tracked, which was confirmed by CT scans. Notably, significant inhibition of tumour growth compared with free Dox was observed in vivo. These findings demonstrated the potential of this hybrid platform as a theranostic tool for tumour treatment.

A study by Gholami et al. explored chitosan-based hybrid platforms loaded with SPIONs and Dox for treating glioblastoma [103]. Drug-release tests demonstrated a rapid release of Dox at pH 5.5, which resembles the pH of the tumour microenvironment, indicating pH-dependent drug-release capability. In addition, the cellular internalization of this hybrid platform was confirmed through fluorescence microscopy. Overall, the study demonstrated the potential of this formulation for both diagnosis and treatment of glioblastoma.

Chen et al. investigated a nanoplatform comprising aptamer-modified graphene QDs and magnetic chitosan for the treatment of hepatocellular carcinoma [105]. It was designed to utilise an aptamer for active targeting and graphene QDs for PTT. In vitro experiments demonstrated the internalization of this hybrid platform in cancer cells and subsequent NIR-triggered drug release. Additionally, the platform showed low cytotoxicity profiles and enhanced accumulation at the tumour site in vivo, which was further validated by imaging. Overall, this system has potential for combined photothermal chemotherapy in cancer treatment.

## 6. Conclusions

This review provides a description and analysis of recent findings in drug delivery and bioimaging applications using hybrid nanoplatforms composed of organic NPs encapsulating inorganic NPs. Findings from these recent investigations suggest that the development of hybrid nanoplatforms holds great potential for targeted cancer therapy and imaging applications. Previous studies have focused on various types of nanocompartments functionalized with different agents for specific biomedical applications. Hybrid nanoplatforms have demonstrated enhanced biocompatibility, stability, efficient drug delivery, improved bioimaging performance, and applicability in different treatment approaches such as PTT, PDT, and US-based. These unique characteristics of hybrid nanoplatforms have enabled successful tumour targeting along with remarkable anticancer outcomes with low side-effect profiles. This demonstrates the potential of hybrid nanoplatforms in improving diagnosis, monitoring, and treatment, allowing new avenues of opportunity for the development of novel therapeutic modalities. 

On the other hand, there is a clear need for the development of effective formulation processes for these hybrid nanostructures. Ongoing challenges relate to (i) the design and optimization of suitable nanostructures, (ii) the controllable production of NPs with desired properties including shape, size, drug loading efficiency, and well-defined drug release or imaging performance, and (iii) scale-up of manufacturing processes for large-scale production. Addressing these challenges requires close collaboration across multiple disciplines, including nanotechnology, chemistry, engineering, and pharmaceutics. In addition, the transition from laboratory-scale research to practical applications in humans requires extensive research to optimize nanostructure formulations, elucidate the underlying mechanisms of action, identify suitable administration routes, and further assess the extent of performance improvement compared with more conventional drug delivery and imaging approaches.

## Figures and Tables

**Table 1:** Liposome-based hybrid platforms for biomedical applications.

Organic NPs	Inorganic NPs	Payload	Application	Target Condition	Highlights	Ref.
Liposomes	AuNPs	Dox	Controlled release/PTT	HeLa and U14 cell line/mice	The platform showed excellent anticancer effects, with up to 78.28% inhibition rate of tumour cells.	[40]
Liposomes	AuNPs	Dox	Controlled release/PTT	A549 cell line	NIR irradiation resulted in >80% drug release within 1 min.	[64]
Liposomes	AuNPs	Fish oil protein (tagged with AuNPs)	Sustained release and targeted delivery	HIG-82 cell line/Osteoarthritic rat model	The first study to report on the anti-osteoarthritic activity of fish oil protein and AuNP encapsulating liposomes.	[65]
Liposomes (conjugated with apo E)	AuNPs	miRNA inhibitors (integrated with AuNPs)	Targeted delivery	U87 cell line/mice	Greater accumulation in brain tumour tissue compared to controls.	[66]
Liposomes (cationic)	AuNPs	Carboplatin	Chemo-radiation therapy	HCT116 cell line/mice	Combination of carboplatin and the hybrid platform was found to be remarkably more efficient in terms of radiosensitization effect.	[67]
Liposomes	Hollow AuNPs	Perfluorocarbon and Dox (stearic acid conjugated)	US-guided fluorescence imaging/PTT	MCF-7, 4T1 and HEK293 cell line/mice	Tumour accumulation of the platform was observed by in vivo fluorescence imaging and the antitumour effect was verified.	[68]
Liposomes	Au Nanorods	Ganoderic acid A	PTT, chemotherapy, antibacterial therapy	*E. coli* and *S. aureus*/MCF-7 cell line/mice	NIR irradiation exhibited broad-spectrum antibacterial effects against drug-resistant *E. coli* and *S. aureus.* Strong anticancer activity was observed against MCF-7 cells.	[69]
Liposomes	Au Nanorods	Ruthenium (II) polypyridyl complexes	Targeted release/PTT	SGC-7901 cell line/mice	NIR irradiation in combination with the nanoplatform could alter the morphology of cells in vitro and could inhibit tumour growth significantly.	[70]
Liposomes	AuNPs-aptamers	Morin	pH-sensitive targeted release	SGC-7901 cell line/mouse	The platform exhibited tumour targeting properties and could inhibit tumour growth.	[71]
Liposomes	Solid AuNPs or hollow AuNPs	Dox	Targeted release/PTT	HDF and MCF-7 cell line/mice	Hollow AuNPs presented eight-fold anticancer efficacy compared with solid AuNPs.	[72]
Liposomes (with FA)	AuNPs and graphene QDs	Dox	Targeted release/bioimaging/PTT/PDT	4T1 and MCF-7 cell line/mice	The platform exhibited in vivo tumour diagnosis capabilities through imaging along with successful PDT.	[73]
Liposomes (folate modified)	Au nanorods and MNPs	Dox	Magnetic and photothermal responsive targeted delivery	5637 and A549 cell line	The hybrid nanoplatform was synthesized using microfluidics-based production; 95% of the drug was released after 3 h.	[74]
Liposomes (with reduced graphene oxide sheets)	Carbon QDs	Dox	Stimuli-sensitive delivery/PTT	MD-MB-231 cell line/mice	Monitoring drug release was accomplished using the emission intensity of the theranostic platform.	[75]
Liposomes	CdSe QDs (modified with oleic acid) and SPIONs (Fe_3_O_4_)	-	Targeted delivery/fluorescence imaging	HepG2 cell line	Magnetic fluorescent liposomes could be drifted by an external magnet that could further be characterized using a fluorescence microscope.	[76]
Liposomes (with RGD peptide)	QDs	L-arginine	Fluorescence imaging-guided PTT	4T1 cell line/mice	The theranostic platform demonstrated the generation of NO, which was toxic to tumour cells in vitro. The accumulation of liposomes in the tumour tissue could be tracked in vivo.	[77]
Liposomes	Graphene QDs	-	US-triggered release	HCT116 cell line	Controlled delivery of QDs (as biomarkers) could be achieved by employing low-frequency US.	[78]
Liposomes (cationic)	CMNPs	-	Magneto-PTT	U87 cell line	The study revealed efficient intracellular uptake of the nanoplatform and exhibited superior hyperthermia effects.	[79]
Liposomes	MNPs	Tenofovir disoproxil fumarate	Multimodal imaging/targeted release	HIV-infected microglia cell line	The platform demonstrated the capability for brain-targeted delivery with assistance of image guidance in vitro.	[62]
Liposomes (ICG loaded and FA modified)	Mesoporous SNPs (with gadolinium)	Dox	PDT/PTT/NIR fluorescence/MRI/PAI	4T1 cell line/mice	The multifunctional theranostic platform demonstrated capability for multimodal imaging, enabled effective diagnostics, and presented Dox release upon NIR irradiation.	[80]
Liposomes (as lipid coating)	Mesoporous SNPs	Berberine	Brain-targeted drug delivery	In vitro assay/mice	The hybrid nanoplatform achieved sustained release of berberine and inhibition of acetylcholine esterase, potentially contributing to the treatment of Alzheimer’s disease.	[81]
Liposomes	AgNPs	-	Drug release analysis	Dialysis bag method	Greater AgNP release was observed at pH 5.5, which corresponds to the pH found in mature endosomes of tumour cells.	[82]
Liposomes	AgNPs	-	Evaluation of cytotoxicity	THP1 cell line	The nanoplatform was found to increase reactive oxygen species-independent induction of apoptosis, suggesting that the encapsulation could potentially reduce the concentration of AgNP required to exert a biological effect.	[83]

NPs: nanoparticles; AuNPs: gold nanoparticles; Dox: doxorubicin; PTT: photothermal therapy; NIR: near-infrared; RT: radiotherapy; QDs: quantum dots; PDT: photodynamic therapy; MNPs: magnetic nanoparticles; US: ultrasound; CMNPs: citric acid-coated iron oxide magnetic nanoparticles; SNPs: silica nanoparticles; MRI: magnetic resonance imaging; PAI: photoacoustic imaging; AgNPs: silver nanoparticles; SPIONs: superparamagnetic iron oxide nanoparticles; NO: nitric oxide; FA: folic acid; ICG: indocyanine green.

**Table 2:** Micelle-based hybrid platforms for biomedical applications.

Organic NPs	Inorganic NPs	Payload	Application	Target Condition	Highlights	Ref.
Micelles (oleic acid and tetraethylene glycol)	AuNPs and IONPs	Dexa	Drug delivery/bioimaging	Dialysis bag	The micellar system was capable of encapsulating Dexa, AuNPs, IONPs, and demonstrated its potential for the delivery of multiple types of therapeutic and diagnostic agents.	[84]
Micelles (polylacticacid stereocomplex)	AuNPs (tethered in the shell)	Dox	PTT/chemotherapy	HepG2 cell line/mice	The nanoplatform was able to provide accelerated drug release via PTE, and showed improved efficacy in tumour reduction.	[85]
Micelles (PHEA-LA-PEG-FA)	Au core (with silica)/QDs shell	Dox	Drug delivery/PTT/bioimaging	MCF7 cell line	The platform was utilised as a theranostic device capable of real-time imaging.	[86]
Micelles	QDs and/or SPIONs	Single stranded DNA (p53) or avidin	Biomolecular detection/tracking	-	The hybrid platform successfully performed rapid, sensitive, and specific separation and detection of DNA and/or protein from a small sample volume.	[87]
Micelles	CuInS_2_/ZnS QDs	-	Intracellular temperature sensing	HeLa and PC3 cell line (mice)	The nanoplatform was efficient in microscale temperature sensing/hyperthermia monitoring through NIR emission with no cytotoxic effect.	[88]
Micelles	CdSe/ZnS QDs	-	Evaluation of toxicity/biosensing	HepG2 cell line	CdSe/ZnS QDs coated with micelles showed minimal toxicity, suggesting that thicker protective polymer layers reduced cytotoxicity and were suitable for bioimaging applications.	[89]
Micelles	SPIONs	Dox	pH-sensitive delivery/bioimaging	HepG2 cell line/mice	The platform successfully achieved drug release and could be imaged through MRI.	[90]

NPs: nanoparticles; AuNPs: gold nanoparticles; Dox: doxorubicin; QDs: quantum dots; IONPs: iron oxide nanoparticles; Dexa: dexamethasone; PTE: photothermal effect; PTT: photothermal therapy; PHEA: α,β-poly(N-hydroxyethyl)-DL-aspartamide; FA: folic acid; SPIONs: superparamagnetic iron oxide nanoparticles; MRI: magnetic resonance imaging; PEG: polyethylene glycol; LA: lipoic acid.

**Table 3:** PLGA-based hybrid platforms for biomedical applications.

Organic NPs	Inorganic NPs	Payload	Application	Target Condition	Highlights	Ref.
PLGA	Hollow Au nanoshell	Anti-PD-1 peptide	Sustained release/PTT	4T1 and CT26 cell line/mice	Efficient PD-1 blocking was achieved through sustained release (for 40 days) of anti-PD-1 peptide by NIR irradiation.	[91]
PLGA	Graphene QDs	Dox	pH-responsive delivery/bioimaging	HeLa cell line	Drug release was observed in a mild acidic environment in vitro. The platform showed its potential for bioimaging applications.	[92]
PLGA	CdSe/ZnS QDs	Chlorophyllin copper complex	PDT	NIH-3T3 cell line	The nanoplatform could generate ROS when excited at 365 nm.	[63]
PLGA (with PEG and Wy5a aptamer)	SPIONs	Docetaxel	Controlled drug delivery/MRI	PC-3 cell line/mice	In vitro investigations demonstrated high-sensitivity MRI detection and enhanced cytotoxic effects. In vivo studies showed that NPs exhibited superior antitumour efficacy while causing minimal systemic toxicity.	[93]
PLGA (with PEG-FA)	SPIONs	Dox	US/MRI/focused US-triggered drug delivery	4T1 cell line/mice	The nanoplatform exhibited enhanced tumour targeting, effective US/MRI contrast, and focused US-triggered drug release.	[94]
PLGA	SNPs (conjugated with Cy7.5)	Docetaxel	Chemo-radiation therapy	Mice	Tracking and sustained drug release from spacers (made of PLGA and loaded with SNPs) were achieved, demonstrating the combined therapeutic efficacy of chemo-radiation therapy.	[95]
PLGA	AgNPs	IFNγ	Cancer therapy	HeLa and MCF-7 cell line	The nanoplatform induced apoptosis through the delivery of AgNPs and IFNγ.	[96]

NPs: nanoparticles; PLGA: poly (lactic-co-glycolic acid); Dox: doxorubicin; PTT: photothermal therapy; PDT: photodynamic therapy; QDs: quantum dots; FA: folic acid; PEG: polyethylene glycol; SPIONs: superparamagnetic iron oxide nanoparticles; IFNγ: recombinant interferon gamma; SNPs: silica nanoparticles; AgNPs: silver nanoparticles; US: ultrasound; MRI: magnetic resonance imaging; ROS: reactive oxygen species.

**Table 4:** Dendrimer-based hybrid platforms developed for use in biomedical applications.

Organic NPs	Inorganic NPs	Payload	Application	Target Condition	Highlights	Ref.
Dendrimers (PAMAM)	Carbon QDs (conjugated with RGDS peptide)	-	Targeted delivery/bioimaging	MDA-MB-231 cell line (for TNBC)	Green synthesis of carbon QDs was successfully performed. The nanoplatform showed potential as a theranostic tool for TNBC, with the capability of detecting and monitoring the presence of Cu (II) ions.	[97]
Dendrimers (conjugated with PEG and Herceptin)	AuNPs	Gadolinium	Targeted delivery/bioimaging	HER-2 overexpressing cell lines	Successful in vitro internalization was achieved with no cytotoxicity. The nanoplatform worked as a nanoimaging agent as well as a nanocarrier for targeted delivery of cytotoxic drugs.	[98]
Dendrimers (PAMAM)	Mesoporous SNPs	Curcumin	Fluorescence imaging/pH-responsive drug delivery	HeLa cell line	The study demonstrated the first-time use of PAMAM dendrimers as pH-sensitive capping and self-fluorescent agents.	[99]
Dendrimer–stabilized Au nanoflowers	Ultrasmall IONPs	-	MRI/CT/PAI-guided combination of PTT and RT	4T1 cell line/subcutaneous tumour model	The multifunctional theranostic platform presented enhanced photothermal conversion efficiency and compatibility with multiple imaging modalities.	[100]

NPs: nanoparticles; AuNPs: gold nanoparticles; QDs: quantum dots; SNPs: silica nanoparticles; IONPs: iron oxide nanoparticles; Dox: doxorubicin; MRI: magnetic resonance imaging; CT: computed tomography; PAI: photoacoustic imaging; PTT: photothermal therapy; RT: radiotherapy; PAMAM: polyamidoamine; TNBC: triple-negative breast cancer; PEG: polyethylene glycol.

**Table 5:** Chitosan-based hybrid platforms developed for usage in biomedical applications.

Organic NPs	Inorganic NPs	Payload	Application	Target Condition	Highlights	Ref.
Chitosan/tripolyphosphate nanogels	Cysteine-functionalized AuNPs	Dox	CT imaging/targeted delivery	OSCC cell line/mice	The hybrid nanogel exhibited high drug-loading capacity (87%) and controlled drug release at acidic pH. AuNPs enabled the monitoring of drug delivery and accumulation in tumours.	[101]
Thermosensitive hydrogel with chitosan	Multiwalled carbon nanotubes	Dox and rhodamine B	Sustained drug delivery/fluorescence imaging	BEL-7402 cell line/mice	Dual drug delivery could be successfully monitored using fluorescence imaging.	[102]
Chitosan	SPION	Dox	Drug delivery/bioimaging	C6 glioma cell line	The nanoplatform could be used as an MRI contrast agent as well as a theranostic tool for glioblastoma.	[103]
Chitosan/alginate	Fe_3_O_4_	Lutein	Magnetic targeting delivery	MDA-MB-231 and MCF-7 cell line	The platform showed enhanced cytotoxicity upon exposure to a magnetic field.	[104]
Magnetic chitosan	Aptamer-modified graphene QDs	Dox	Photothermal chemotherapy	Hepatoma cell line H22/mice	There was no evidence of substantial biological toxicity or adverse effects in either in vivo or in vitro experiments.	[105]
Chitosan	Mesoporous SNP	Dox and indocyanine green	Chemotherapy/PDT	HepG2 cell line	The platform could successfully target and kill cells via chemotherapy combined with PDT.	[106]

NPs: nanoparticles; AuNPs: gold nanoparticles; Dox: doxorubicin; CT: computed tomography; SPIONs: superparamagnetic iron oxide nanoparticles; MRI: magnetic resonance imaging; QDs: quantum dots; SNPs: silica nanoparticles; PDT: photodynamic therapy.

## Data Availability

Not applicable.
